# Aortic Valve Stenosis: Progress from Diagnosis to Treatment

**DOI:** 10.3390/jcm15020659

**Published:** 2026-01-14

**Authors:** Paolo Ossola, Simone Ghidini, Elena Gualini, Francesca Daus, Francesco Politi, Claudio Ciampi, Roberto Spoladore, Francesco Musca, Alessandro Maloberti, Cristina Giannattasio

**Affiliations:** 1 Cardiology Department De Gasperis Cardio Center, Niguarda Hospital, 20162 Milan, Italy; elena.gualini@ospedaleniguarda.it (E.G.); francesca.daus@ospedaleniguarda.it (F.D.); francesco.politi@ospedaleniguarda.it (F.P.); francesco.musca@ospedaleniguarda.it (F.M.); alessandro.maloberti@ospedaleniguarda.it (A.M.); cristina.giannattasio@ospedaleniguarda.it (C.G.); 2 Istituto di Ricovero e Cura a Carattere Scientifico (IRCCS), Istituto Auxologico Italiano, Department of Cardiology, Cardiac Rehabilitation Unit, S. Luca Hospital, Piazzale Brescia 20, 20149 Milan, Italy; s.ghidini@auxologico.it; 3 UOC Cardiologia, Ospedale Garibaldi-Nesima, Azienda di Rilievo Nazionale e Alta Specializzazione ‘Garibaldi’, 95122 Catania, Italy; c.ciampi1@campus.unimib.it; 4 Heart Failure Clinic, Division of Cardiology, Alessandro Manzoni Hospital, ASST Lecco, 23900 Lecco, Italy; ambulatorio.rscardiologia@gmail.com

**Keywords:** aortic stenosis, transcatheter aortic valve replacement, surgical aortic valve replacement, amyloidosis, heart failure, cardiogenic shock, echocardiography, degenerative, calcification

## Abstract

Aortic stenosis (AS) is the most prevalent valvular heart disease in Western countries and it is especially associated with older age. With its progressive course, AS leads to ventricular hypertrophy, impaired diastolic and systolic function, and symptomatic deterioration. The natural history of AS is closely linked to the extent of myocardial and extracardiac damage in association with the patients comorbidities. Diagnosis relies primarily on transthoracic echocardiography, which assesses valve morphology, quantifies stenosis severity, and evaluates cardiac remodeling. However, discordant grading is frequent, necessitating advanced imaging to clarify the severity and the mechanism of the stenosis and stratify risk. Treatment is predominantly interventional, as no medical therapy is able to stop disease progression. Surgical aortic valve replacement (SAVR) and transcatheter aortic valve replacement (TAVR) are the two treatment options. Special clinical scenarios—such as cardiogenic shock or concomitant cardiac amyloidosis—pose additional diagnostic and therapeutic challenges and require individualized, multidisciplinary management. Overall, contemporary AS care increasingly integrates multimodality imaging, refined risk stratification, and tailored interventional strategies to optimize outcomes.

## 1. Introduction

Aortic stenosis (AS) is a chronic and progressive disorder and represents the most prevalent valvular heart disease in Western countries [1]. Its incidence and prevalence increase steadily with the aging of the general population, with prevalence rates ranging from 5% to 12% among individuals older than 75 years in several epidemiologic studies [2,3].

In developed countries, the degenerative calcific form of the tricuspid valve is the most common etiology. In contrast, congenital bicuspid aortic valve disease remains the leading cause of AS in younger patients, often resulting in earlier onset and more rapid disease progression [2].

In the past, the treatment of severe symptomatic AS relied exclusively on surgical aortic valve replacement. Over the past few decades, however, a percutaneous approach using TAVR has gained widespread adoption, profoundly changing the therapeutic landscape of this condition [1,4,5].

In this narrative review, we discuss the current state-of-the art on pathophysiology, progression, clinical implication, and management of severe aortic stenosis.

## 2. Epidemiology

AS is a highly prevalent valvular heart disease worldwide, with a significant impact on physical function, quality of life, and overall survival in both sexes [6,7]. No substantial sex-related differences have been observed in the general population; however, the proportion of affected women increases progressively in the oldest age groups. Women are more frequently represented among the very elderly and tend to present with greater clinical severity at the time of diagnosis. This pattern may reflect a combination of factors, including delayed recognition, subtler symptom onset, smaller ventricular dimensions, and potential differences in referral and diagnostic practices [7,8].

Epidemiological data indicate that more than one quarter of adults over the age of 65 in Western countries exhibit some degree of aortic valve sclerosis, which may progressively evolve into clinically significant AS. The annual incidence of clinically significant aortic stenosis in Europe is estimated to range from 4 to 7 cases per 1000 person-years among individuals older than 75 years [7,9]. Milder stages of the disease often progress to severe obstruction, which, when left untreated, is associated with a poor prognosis, with a five-year event-free survival rate below 40% [10,11]. When AS becomes severe and symptomatic (with the development of angina, syncope, or heart failure), median survival decreases dramatically to approximately two to five years [12], accounting for more than 100,000 deaths worldwide each year [11,13,14].

## 3. Etiology and Pathophysiology

The etiologies of AS in the general population are mainly two and vary according to age [2]. In younger patients, the predominant cause is congenital, most commonly a bicuspid aortic valve [15]. This condition, characterized by a reduced number of cusps, leads to abnormal hemodynamics and may progress to aortic stenosis, regurgitation, or both [16]. Bicuspid aortic valve is a frequent congenital defect, with a prevalence of 1–2% and is associated with an inherited predisposition and incomplete penetrance [17]. The morphological abnormality alone is insufficient to cause disease; environmental factors contribute to valve calcification and gradient progression [16]. Asymmetric cusp motion increases shear stress, favoring calcium deposition in regions distinct from those affected in degenerative AS, ultimately resulting in valve restriction and often associated dilation of the aortic root and ascending aorta [16,18].

In elderly patients, AS is most commonly degenerative and affects tricuspid aortic valves [17]. Risk factors overlap with those of atherosclerosis and coronary calcification [2,19], promoting oxidative stress and inflammation. This microenvironment activates bone morphogenetic protein-2 (BMP-2), driving valvular interstitial cells toward an osteoblastic phenotype and leading to progressive calcification from early lesions to overt stenosis [19]. Less common etiologies include rheumatic disease, radiotherapy-related injury, and rare metabolic disorders observed in pediatric populations (e.g., ochronosis, alkaptonuria) [2]. Elevated lipoprotein(a) [Lp(a)] is strongly associated with aortic valve calcification and stenosis by promoting oxidative inflammation and osteogenic pathways. While high Lp(a) levels predict disease onset and faster progression, their influence appears to be greater during the early stages of AS [20].

**Figure 1:**
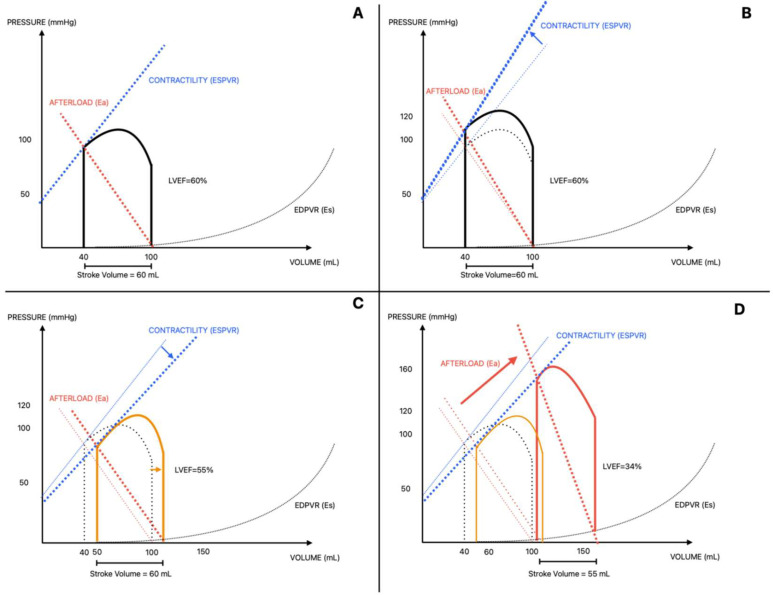
PV loops illustrating the adaptive mechanisms observed in the presence of aortic stenosis: (**A**) Normal PV loop. (**B**) The increase in afterload is compensated by an increase in contractility (hypertrophy). (**C**) When further hypertrophy is no longer possible, the ventricle utilizes the preload reserve by increasing LVEDV; in this way, afterload remains unchanged. (**D**) Once the Frank-Starling compensatory mechanism reaches its limit, the preload reserve is exhausted, afterload increases, and systolic dysfunction develops, leading to a reduction in stroke volume. Ea: arterial elastance; EDPVR: end diastolic pressure–volume relationship; Es: ventricular elastance; ESPVR: end systolic pressure–volume relationship; LVEF: left ventricle ejection fraction.

Regardless of etiology, AS ultimately leads to a common hemodynamic consequence characterized by an increased transvalvular gradient and left ventricular pressure overload. This chronic increase in afterload drives ventricular remodeling. Conceptually, afterload can be described by Laplace’s law, whereby wall stress increases with intraventricular pressure and chamber radius and decreases with wall thickness [21]. In response, concentric hypertrophy develops to preserve wall stress and cardiac output. Ross et al. described three stages of disease progression, with partial overlap between the first two [22]. Initially, pressure overload induces adaptive hypertrophy with preserved or increased systolic function (Figure 1B) [22]. Over time, hypertrophy becomes maladaptive, leading to impaired diastolic function, increased myocardial stiffness, and reduced perfusion with depletion of coronary flow reserve [21,23]. These mechanisms may contribute to angina, although symptoms correlate poorly with the degree of hypertrophy or valvular obstruction [21].

In more advanced stages, compensatory mechanisms fail: ventricular dilation and increased end-diastolic volume initially maintain stroke volume through the Frank–Starling mechanism (Figure 1C) [22]. However, progressive exhaustion of preload reserve results in reduced stroke volume and a marked increase in afterload (Figure 1D). This condition, termed afterload mismatch, reflects the inability of the ventricle to sustain adequate output against excessive afterload and is often associated with myocardial fibrosis and reduced contractility, suggesting limited reversibility despite valve intervention [22,24]. Clinically, ventricular dysfunction and increased afterload manifest as heart failure symptoms such as dyspnea and congestion. Syncope is less clearly explained and typically occurs during exertion; it has been hypothesized to result from reduced cardiac output reserve combined with an inappropriate fall in systemic vascular resistance, although this mechanism remains partly speculative [21].

## 4. Progression and Classification

The pathophysiological process described above parallels the clinical course of patients with aortic stenosis. It is important to emphasize that, although the disease follows a progressive pattern, not all patients develop severe or symptomatic stenosis, and the rate of progression increases in proportion to the severity of obstruction [12]. The natural history of the disease is reviewed below according to the 2020 American guidelines classification [25], highlighting the main clinical entities and their related therapeutic implications (Table 1).

The first phase corresponds to the “at-risk” stage (Stage A), during which, through a complex interplay between non-modifiable factors (e.g., bicuspid aortic valve) and environmental influences (leaflet degeneration), cusp sclerosis, and subsequently mild stenosis develop. Quantifying the incidence of this phenomenon is challenging due to multiple evaluation biases. Population-based registry data provide some insight: for example, the Swedish registry [26] reports an age-adjusted incidence of 11.4 per 100,000 men and 7.1 per 100,000 women in the early 2000s, although this likely underestimates mild forms, as only moderate-to-severe stenoses were fully captured. Similarly, the Quebec registry [27] reported an age-adjusted incidence of 1.2%, a figure that is also likely underestimated because it included only hospitalized patients.

Mild and moderate stages of stenosis fall under “progressive aortic stenosis” (Stage B). The systematic review and meta-analysis by Willner et al. [12] evaluated echocardiographic parameters in patients with aortic stenosis of various etiologies (bicuspid valve reported in 10 of 20 studies, with a prevalence of 12%). They found an annual increase of 0.09 m/s in peak aortic jet velocity and 2.3 mmHg in mean gradient among patients with mild stenosis. In the landmark 1997 study by Otto et al. [28], 123 patients with aortic stenosis (mean age 61 years) were prospectively followed. Patients with mild stenosis at baseline (velocity < 3 m/s) exhibited an excellent prognosis, with a two-year survival of 84 ± 16%, at a time when transcatheter valve replacement therapies were not yet available. Progression of moderate stenosis occurred more rapidly (mean gradient increase 4–6 mmHg/year and peak velocity increase 0.17–0.24 m/s/year according to Willner et al.), although two-year survival remained relatively preserved (66 ± 13%) [12,28].

Once thresholds for severe stenosis are reached, further acceleration of gradient increase and valve narrowing is observed (7 mmHg/year and 0.28 m/s/year, respectively) [12], accompanied by a marked deterioration in survival (only 21% without intervention in Otto’s cohort) [28]. Prognosis worsens progressively with increasing maximum transvalvular velocity, as demonstrated by other studies [11]. However, the literature has increasingly emphasized the need to better stratify patients with severe aortic stenosis using additional parameters independent of stenosis severity alone [2,15].

The first parameter to consider is clinical presentation. The pathophysiological changes previously described reach maximal compensation in patients who meet criteria for severe stenosis but remain asymptomatic (Stage C1). These patients generally have an acceptable prognosis [13,15,29], although disease progression is almost inevitable, with approximately one-third developing symptoms within two years [30]. Impaired coronary perfusion, elevated filling pressures, and contractile dysfunction are the main mechanisms leading to the development of chest pain, syncope, and dyspnea. As early as 1968, Ross et al. [31] identified the prognostic relevance of symptom type in aortic stenosis (Stage D1): angina and syncope were associated with an average survival of three years, compared with two years for dyspnea and one year for heart failure [31]. The worse prognosis associated with heart failure reflects the development of systolic dysfunction and the breakdown of previously effective compensatory mechanisms. Impaired Frank–Starling coupling and afterload mismatch lead to increased afterload, contractile dysfunction, and progressive myocardial fibrosis. At this stage, even valve replacement may be performed too late to achieve full recovery [22,24,32] (Stage D2, commonly referred to as “low-flow, low-gradient” aortic stenosis).

The natural history is further complicated by marked heterogeneity in patient presentation. Patients with severe asymptomatic stenosis and systolic dysfunction (Stage C2) exhibit a worse prognosis than those with preserved systolic function [29]. Conversely, others may present with symptomatic severe stenosis, low gradients, and preserved systolic function (Stage D3, also known as paradoxical low-flow aortic stenosis). These patients are typically elderly, hypertensive individuals with small, hypertrophic ventricles, whose prognosis resembles that of patients with Stage B disease [33].

Overall, the natural history of aortic stenosis demonstrates that prognosis depends less on the valvular lesion itself, considered in isolation, and more on the secondary hemodynamic and cardiac alterations it induces. Based on this concept, a new classification has been proposed (Table 2) [19,34], in which severe aortic stenosis is viewed as a progressive process with progressively higher mortality, even after aortic valve replacement [34].

## 5. Diagnosis

Accurate diagnosis of AS is essential for appropriate clinical decision-making and optimal timing of intervention. The diagnostic work-up integrates clinical evaluation with multimodality imaging.

Transthoracic echocardiography (TTE) represents the first-line, and most informative imaging technique for the diagnostic evaluation of AS. In these patients, it serves three main purposes: confirming the presence and characterizing the morphology of the aortic valve; quantifying the severity of stenosis; and evaluating the structure and function of the left ventricle, other cardiac chambers, and the aorta [35]. Assessment of AS severity relies on a comprehensive hemodynamic evaluation using key quantitative parameters, including peak aortic jet velocity, mean transvalvular pressure gradient, and aortic valve area (AVA) calculated using the continuity equation [36]. However, discordant findings among these parameters are not uncommon and require careful interpretation of flow conditions and measurement accuracy.

The dimensionless velocity index (DVI), particularly useful in unclear cases, provides a flow-independent parameter that supports severity grading, especially when left ventricular outflow tract (LVOT) measurements are uncertain. Assessment of stroke volume index (SVI) is also crucial, as low-flow states (SVI < 35 mL/m^2^) may lead to underestimation of AS severity despite significant obstruction [37,38].

Up to 40% of patients with severe AS show apparent discordance between AVA and transvalvular flow parameters. In most cases, AVA suggests severe stenosis, whereas peak velocity or mean gradient indicate only moderate obstruction. This discordant grading encompasses three main hemodynamic subsets:

- Low-flow, low-gradient AS, characterized by SVI < 35 mL/m^2^ and reduced left ventricular ejection fraction (LVEF < 50%);
- Paradoxical low-flow, low-gradient AS, defined by SVI < 35 mL/m^2^ despite preserved LVEF (≥50%);
- Normal-flow, low-gradient AS, with SVI ≥ 35 mL/m^2^ and preserved LVEF (≥50%).

Among these entities, classical low-flow, low-gradient AS represents the greatest diagnostic challenge, as it may reflect either true severe stenosis or pseudo-severe obstruction secondary to left ventricular systolic dysfunction. In this setting, dobutamine stress echocardiography (DSE) is a fundamental diagnostic test. During incremental low-dose dobutamine infusion, true severe AS is identified when AVA remains ≤1.0 cm^2^ while transaortic velocity and/or mean pressure gradient increase. Conversely, a marked increase in AVA with minimal change in gradient suggests pseudo-severe disease.

The absence of contractile (flow) reserve—defined as a ≥20% increase in stroke volume during DSE—provides important prognostic information, as it is associated with higher perioperative risk and poorer long-term outcomes after aortic valve intervention. Nonetheless, emerging evidence indicates that in patients without contractile reserve, computed tomography (CT) calcium scoring may provide complementary information to confirm true AS severity and guide management decisions [39,40].

Transoesophageal echocardiography (TEE) plays a complementary role, primarily when TTE is suboptimal or when detailed anatomical characterization is required. TEE is particularly valuable for high-resolution imaging of valve morphology, leaflet number, mobility, and calcification, as well as for direct planimetric measurement of AVA. However, Doppler-derived hemodynamic measurements obtained by TEE may differ from those obtained by TTE due to altered loading conditions and insonation angles. Therefore, TEE-derived gradients and AVA should be interpreted with caution and in conjunction with clinical and TTE findings [41,42].

Moreover, global longitudinal strain (GLS), as evaluated by echocardiography or CMR, is a sensitive and quantitative marker of subclinical left ventricular dysfunction, as it reflects the degree of longitudinal fiber shortening and is particularly affected by pressure overload, concentric remodeling and myocardial fibrosis. 

Impaired GLS is independently associated with increased risk of symptom development, adverse cardiac events, and mortality in both moderate and severe AS, even when LVEF is preserved, with a suggested threshold for risk stratification < −14.7% according to a recent metanalysis [43,44]. Additionally, a GLS value of <−9.7% is associated with significantly higher 1- and 5-year mortality in AS patients treated conservatively [45].

Consequently, this may prompt consideration of AVR before the onset of overt symptoms or LVEF decline.

With regard to functional capacity assessment, exercise testing is indicated to objectively unmask symptoms and abnormal blood pressure responses that may not be apparent from clinical history alone. The development of symptoms or an abnormal blood pressure response during exercise is considered an indication for aortic valve intervention. Exercise testing is contraindicated in symptomatic patients with severe AS because of the risk of severe hemodynamic compromise, including syncope and arrhythmias [46].

Cardiopulmonary exercise testing (CPET) is recognized as a valuable adjunct for risk stratification in selected cases, particularly for quantifying peak oxygen consumption and ventilatory efficiency, but it is not recommended for routine assessment [47].

While echocardiography remains the first-line modality for diagnostic confirmation and functional assessment of AS, certain clinical scenarios require additional imaging evaluation. In this context, computed tomography (CT) and cardiac magnetic resonance (CMR) have emerged as complementary techniques that provide anatomic, functional, and tissue characterization beyond the capabilities of echocardiography [35,48].

CT is particularly useful for quantifying aortic valve calcification, which correlates with AS severity and is especially helpful in cases of discordant echocardiographic grading. Calcium scoring provides a reliable assessment of valve calcification, aiding in the differentiation between severe and non-severe AS when Doppler findings are inconclusive. CT is also essential for pre-procedural planning of transcatheter aortic valve replacement (TAVR), providing accurate measurements of annular dimensions, coronary ostia height, and vascular access [48].

CMR provides additional valuable information, especially when echocardiographic and CT findings are inconclusive or when further myocardial characterization is required. It allows for accurate and reproducible quantification of left ventricular volumes, mass, and function, which is essential for assessing AS-related remodeling and for longitudinal follow-up. In addition, CMR enables direct visualization of the aortic valve and root, and AVA planimetry by CMR has been shown to be equivalent to TEE [48,49].

From a functional perspective, CMR employs phase-contrast velocity mapping, including four-dimensional (4D) flow techniques, to quantify transvalvular flow, pressure gradients, and regurgitant volumes. This provides a valuable alternative when Doppler echocardiography is limited or discordant with clinical findings, particularly in low-flow, low-gradient AS or in the presence of complex LVOT geometry [50,51].

Notably, the unique strength of CMR lies in tissue characterization. Both focal replacement fibrosis, detected by late gadolinium enhancement (LGE), and diffuse interstitial fibrosis, quantified by extracellular volume fraction (ECV%) or T1 mapping, are independently associated with increased all-cause mortality, cardiovascular mortality, and heart failure events in AS. Patients with moderate AS or asymptomatic severe AS who exhibit elevated ECV% or LGE have significantly worse outcomes, demonstrating incremental prognostic value beyond standard clinical and echocardiographic parameters and enabling more refined risk stratification and identification of individuals who may benefit from closer surveillance or earlier valve intervention [52,53].

Furthermore, observational data suggest that diffuse interstitial fibrosis may partially regress following aortic valve replacement and relief of pressure overload, contributing to reverse remodeling and improved clinical outcomes. In contrast, focal fibrosis reflects irreversible myocardial injury, and its preoperative presence is associated with poorer outcomes and limited functional recovery after intervention [24,54].

## 6. Treatment

The management of aortic stenosis (AS) has changed substantially over the past 15 years and continues to evolve considering emerging evidence and updated clinical practice guidelines, particularly the 2025 European Society of Cardiology (ESC) recommendations. The cornerstone indication for intervention remains severe symptomatic AS, including low-flow, low-gradient forms with reduced LVEF and paradoxical low-flow AS with preserved ejection fraction, given the unfavorable natural history of the disease.

According to the 2025 ESC Guidelines, intervention is also indicated in asymptomatic severe AS in the presence of left ventricular systolic dysfunction (LVEF ≤ 50%) without alternative causes [1], or in patients with severe valve calcification (cAVCS > 2000 in men, >1200 in women) and rapid disease progression (V_max_ ≥ 0.3 m/s/year). As no medical therapy has demonstrated efficacy in slowing AS progression, treatment is primarily interventional, either by surgical aortic valve replacement (SAVR) or transcatheter aortic valve replacement (TAVR). Palliative medical therapy should be reserved for patients with very limited life expectancy or in the presence of severe comorbidities precluding meaningful clinical benefit. Several recent trials (EARLY TAVR, AVATAR, EVOLVED) have explored the role of early AVR vs. clinical surveillance in asymptomatic severe AS. In the EARLY TAVR trial, early intervention significantly reduced the composite endpoint of death, stroke, or unplanned cardiovascular hospitalization compared with surveillance [55]. However, this benefit was largely driven by a reduction in hospitalizations primarily due to the early onset of AS-related symptoms within six months after randomization, while no significant differences were observed in all-cause mortality or stroke. These findings support a more cautious interpretation of early TAVR, emphasizing patient selection rather than a universal early-intervention strategy.

The EVOLVED trial investigated early AVR in patients with myocardial fibrosis detected by LGE, based on the hypothesis that fibrosis may represent an early marker of ventricular decompensation. However, the study was underpowered due to recruitment limitations during the COVID-19 pandemic and failed to meet its composite endpoint of mortality or unplanned AS-related hospitalization [56], limiting definitive conclusions regarding this strategy.

Long-term follow-up of the AVATAR trial demonstrated a reduction in a composite endpoint including mortality and heart failure hospitalization with early SAVR, although the sample size was small [57]. Consistently, a meta-analysis of these trials did not demonstrate a mortality benefit of early AVR but confirmed a reduction in heart failure hospitalizations and stroke [58]. Overall, these data suggest that early intervention may be considered in selected asymptomatic patients with severe, high-gradient AS and preserved LVEF when procedural risk is low, but this remains a Class IIa recommendation supported by Level of Evidence A [1].

Importantly, decisions regarding timing and type of intervention should incorporate lifetime management considerations from the outset, including prosthesis durability, feasibility of redo procedures, and future coronary access. Multidisciplinary Heart Team evaluation remains essential, integrating life expectancy, comorbidities, prosthesis durability, valve anatomy, and patient preferences.

The ESC age threshold favoring TAVR has been lowered from 75 to 70 years, reflecting long-term data demonstrating non-inferiority of TAVR compared with SAVR, not only in high- and intermediate-risk populations, as it was at the beginning of the “percutaneous era” but also in low-risk patients [1,59]. Ten-year follow-up of the NOTION trial showed comparable rates of mortality, stroke, and myocardial infarction between TAVR and SAVR [60], with similar findings at 5 years in the PARTNER 3 and Evolut Low Risk trials [61,62]. Comparable results were reported in the NOTION 2 trial, which included younger patients (mean age 71.1 yrs old) albeit with shorter follow-up and smaller sample size [63]. By contrast, the 2020 ACC/AHA Guidelines, already, adopted an age cutoff for SAVR of 65 years, primarily based on U.S. actuarial life expectancy tables and the balance between projected patient longevity and valve durability. Either SAVR or TAVR is considered appropriate for patients aged 65–80 years, depending on a case by case evaluation. Heart Team evaluation was, indeed, already strongly recommended in the 2020 American Guidelines. TAVR is always suggested in patients of at least 80 years old [25].

Beyond short-term outcomes, contemporary decision-making must increasingly adopt a lifetime management perspective. The durability of the first implanted prosthesis, the feasibility of future redo procedures (valve-in-valve versus redo sternotomy), and the preservation of coronary access are critical determinants of long-term outcomes. Advances in bioprosthetic durability and valve-in-valve strategies have expanded treatment options [64], but suboptimal initial valve choice or sizing may increase the risk of patient–prosthesis mismatch, coronary obstruction, or limited coronary access in the future [65,66,67]. Therefore, the selection of the initial therapeutic approach should not be viewed as an isolated decision but rather as a pivotal step that directly influences a patient’s long-term clinical trajectory, procedural options, and quality of life [68,69,70,71,72,73].

## 7. Clinical Scenario

### 7.1. Clinical Scenario: Cardiogenic Shock

Approximately 10–24% of patients admitted for AS undergo urgent or emergency treatment, and 1.6–3.2% of patients undergoing TAVR present with cardiogenic shock (CS) [74,75]. Acute decompensation is often triggered by mechanisms such as acute myocardial ischemia, tachyarrhythmias, or sudden volume shifts, which are poorly tolerated due to the fixed afterload and reduced left ventricular (LV) compliance secondary to elevated LV end-diastolic pressure (LVEDP) and concentric LV hypertrophy [73].

Tachyarrhythmias, particularly sinus tachycardia, are usually compensatory to maintain stroke volume, but rapid atrial fibrillation can worsen hemodynamics by reducing atrial contribution to LV filling, increasing left atrial pressure, pulmonary venous congestion, and post-capillary wedge pressure [76]. Heart rate control may be achieved with ultra-short-acting agents such as landiolol [77] or, alternatively, digoxin or electrical cardioversion in unstable patients [74]. Diuretic therapy should be initiated to optimize volume status, guided by careful clinical and hemodynamic assessment [78]. Inotropic support (dobutamine, norepinephrine, epinephrine) can be considered when necessary [79,80,81], while vasodilators (e.g., nitroprusside) are generally avoided due to the risk of hypotension and worsened transvalvular gradients [82].

CS in severe AS presents a complex scenario, often requiring urgent treatment of the underlying valvular pathology. Mechanical support devices such as the Impella are generally contraindicated in severe aortic stenosis or insufficiency, although selected cases may benefit under specialized conditions [75,83,84]. In practice, TAVR and balloon aortic valvuloplasty (BAV) are preferred over surgical AVR due to high perioperative mortality [85]. BAV, recommended as a bridge to TAVR or SAVR in patients with CS [74,75], carries a risk of restenosis, mortality, and acute aortic regurgitation [86,87,88,89,90,91], and is therefore mainly reserved for palliative care or bridge-to-decision strategies in high-volume centers [89].

TAVR offers more durable outcomes and has been shown to be feasible in emergency settings, though mortality remains higher than elective procedures, primarily due to the severity of CS rather than procedural complications [75,84]. Device success rates in CS, as defined by VARC criteria, vary between 84.2% and 100% [92,93,94]. Despite advances, management of CS due to AS remains challenging, with limited high-quality evidence, and large prospective RCTs on emergency TAVR in this population are lacking.

### 7.2. Clinical Scenario: Cardiac Amyloidosis

Concentric left ventricular hypertrophy associated with aortic stenosis is often not solely the result of chronically increased afterload on the ventricular chamber, but may also represent the manifestation of an infiltrative disorder such as amyloidosis, characterized by the deposition of misfolded proteins. The combination of AS and CA complicates the diagnosis and therapeutic management of both conditions. Transthyretin cardiac amyloidosis (ATTR-CA) is, by far, the disease phenotype most frequently associated with aortic stenosis. Recent data indicate that it affects between 50,000 and 150,000 individuals in the United States (incidence 12.1 cases), with a median survival of approximately five years in wild-type patients [95]. The average age of CA patients ranges from 74 to 90 years, with a median age of 66 years, even if most diagnoses occurring in men in their eighth or ninth decade [96,97]. ATTR-CA produces a restrictive physiology, low flow haemodynamics and symptoms that are often disproportionate to valvular metrics; clinical “red flags” (carpal tunnel syndrome, lumbar spinal stenosis, biceps tendon rupture, autonomic dysfunction), EKG features (low voltage relative to LV mass, pseudo infarct patterns), and echocardiographic clues (apical sparing strain pattern) should prompt targeted evaluation with bone scintigraphy and serum/urine immunofixation because the diagnosis alters prognosis, may attenuate symptomatic gains after AVR and opens disease specific therapeutic options that must be coordinated with valve management. The coexistence of AS and ATTR-CA confers a poor prognosis, with increased incidence of heart failure, arrhythmias, cardiomegaly, carpal tunnel syndrome, chronic kidney disease, and peripheral neuropathy [98,99,100].

Annabi et al. [98] further support these findings by showing that aortic stenosis is highly prevalent in patients with wild-type ATTR amyloidosis (ATTRwt), affecting 26% of this group, compared to much lower rates in hereditary ATTR (8%) and light-chain amyloidosis (5%). The predominant echocardiographic pattern in patients with cardiac amyloidosis and severe AS is low-flow, low-gradient AS, which is associated with worse outcomes and diagnostic challenges [95,100,101].

In addition to the well-established myocardial injury characterized by the classic ‘apical sparing’ pattern, the infiltration of misfolded proteins induces a state of chronic inflammation and stiffness, creating a profibrotic environment that leads to damage of both the aortic valvular apparatus and the cardiac conduction system [100,102,103]. In this clinical context, beta-blocker therapy, although indicated, may be poorly tolerated due to the increased risk of bradyarrhythmias and concomitant autonomic dysfunction. This is particularly relevant in elderly patients and those with advanced myocardial remodeling, where impaired conduction and autonomic instability are common, and careful titration or avoidance of beta-blockers may be necessary. Similarly, instability of fluid balance complicates diuretic titration, especially in patients with markedly remodeled ventricles. Loop diuretics remain the mainstay for congestion management in heart failure, but dosing must be individualized to avoid hypovolemia, electrolyte disturbances, and renal dysfunction. Whene the AS become severe, the surgical treatment options are TAVR or SAVR, depending on patient characteristics [104,105], with similar outcomes between TAVR and SAVR over a median follow-up of 15.3 months [106].

The findings from Masri et al. (2025) demonstrate that patients with both ATTR—CA and AS experience significantly higher risks of mortality and heart failure hospitalization compared to those with AS alone [107]. Specifically, the hazard ratio for death was 1.3 and for heart failure hospitalization was 1.9 in the dual pathology group, underscoring the adverse prognostic impact of this coexistence. Even after aortic valve replacement, mortality remained elevated in patients with both conditions, indicating that valve intervention alone does not fully mitigate risk in this population.

## 8. Discussion

Multimodality imaging has reshaped contemporary management of aortic stenosis [108]. This integrated perspective is essential because discordant presentations (for example, a small AVA with low gradient) are common and require a reproducible, image driven pathway to avoid both undertreatment of true severe disease and unnecessary intervention for pseudo severe obstruction [109].

In routine practice, meticulous transthoracic echocardiography remains the indispensable first step with an exhaustive and standardized measurements of LVOT, Doppler waves and routine calculation of stroke volume index [109]. When Doppler grading remains discordant despite rigorous technique, complementary functional and anatomic tests are pragmatic and often decisive [108] like low dose dobutamine stress echocardiography to assess contractile (flow) reserve. When DSE is not feasible or inconclusive, non-contrast multidetector CT quantification of aortic valve calcification (AVC) provides a low independent anatomic anchor [108].

Risk stratification should extend beyond valve-related parameters to include myocardial phenotyping, as the extent and pattern of myocardial injury influence both short-term procedural risk and long-term functional recovery. Deformation imaging (global longitudinal strain), mitral annular S’, myocardial contraction fraction, and natriuretic peptides can detect subclinical systolic dysfunction not captured by LVEF alone, thereby providing incremental prognostic information [110].

In asymptomatic aortic stenosis, reduced GLS is associated with a higher risk of symptom development, adverse cardiac events, and the need for valve intervention. GLS deterioration precedes symptom onset and LVEF decline, and its incorporation into risk stratification may help identify patients who could benefit from earlier intervention [111,112,113]. The integration of strain parameters and CMR markers into preprocedural assessment helps identify the “vulnerable ventricle” and supports optimal timing of intervention before irreversible myocardial scarring develops [19,114].

Framing AS as a valve–myocardium–pulmonary vascular syndrome clarifies why some patients with borderline valvular metrics nonetheless experience poor outcomes and therefore may benefit from earlier intervention when extracardiac sequelae indicate limited reversibility [19].

Randomized trials and biomarker guided strategies have begun to test whether earlier AVR improves clinically meaningful outcomes in selected asymptomatic patients; contemporary randomized studies and CMR or biomarker driven approaches report consistent reductions in composite endpoints—driven largely by fewer heart failure admissions, fewer unplanned cardiovascular hospitalizations and improved quality of life—when intervention is performed before overt symptom onset in carefully selected cohorts [112]. However, mortality benefits are heterogeneous across trials and depend critically on patient selection, the burden of irreversible myocardial fibrosis, procedural risk and the competing harms of premature prosthesis implantation (durability, prosthesis patient mismatch, endocarditis and anticoagulation burden) [112]. These data therefore support a personalized-approach. Choice of procedural modality requires multidisciplinary Heart Team discussion with the integration of anatomic suitability, coronary revascularization needs, frailty and comorbidity [113].

A pragmatic and increasingly important special consideration is the coexistence of ATTR-CA in older AS cohorts [97]. ATTR-CA produces a restrictive physiology, low flow haemodynamics and symptoms that are often disproportionate to valvular metrics, and in this context the prompt diagnosis is fundamental [97].

In summary, contemporary management of AS must be predicated on an integrated, multimodality approach that treats the disease as a valve–myocardium–pulmonary vascular syndrome rather than a solitary valvular lesion. Embedding myocardial phenotyping, cardiac damage staging and validated anatomic biomarkers into routine pathways will improve patient selection, optimize timing of intervention, and align procedural choices with long term patient trajectories. Future research priorities include long term comparative outcomes of early AVR strategies, robust biomarkers that discriminate reversible from irreversible myocardial injury, and pragmatic trials that test imaging driven algorithms against patient centered endpoints.

## 9. Conclusions

Aortic valve stenosis is a progressive, multisystem disease that requires timely diagnosis, precise phenotyping and lifetime-oriented management. High quality transthoracic echocardiography remains the diagnostic cornerstone; when Doppler findings are discordant, a prespecified escalation to functional testing, CT calcium scoring and CMR tissue characterization should be applied. Risk stratification must move beyond LVEF to include deformation imaging, biomarkers, and CMR indices that distinguish reversible from irreversible myocardial injury and inform timing of intervention. Symptomatic severe AS mandates prompt AVR; selected asymptomatic patients with imaging or biomarker evidence of imminent decompensation may benefit from earlier intervention after multidisciplinary Heart Team evaluation. Choice between SAVR and TAVR should be individualized with explicit lifetime planning for prosthesis durability and future reinterventions. Routine screening for comorbid conditions that alter prognosis (for example transthyretin cardiac amyloidosis) and centralized care in heart valve clinics with standardized protocols will improve consistency of decision making. Priority research goals are validation of imaging/biomarker thresholds for reversibility, and pragmatic trials comparing imaging driven early intervention strategies with long term, patient centered outcomes.

## Figures and Tables

**Table 1:** Stages of AS. AR indicates aortic regurgitation; AS, aortic stenosis; AVA, aortic valve area circulation; AVA_i_, AVA indexed to body surface area; BAV, bicuspid aortic valve; ΔP, pressure gradient between the LV and aorta HF, heart failure; LV, left ventricular; LVEF, left ventricular ejection fraction; and V_max_, maximum velocity. Adapted from Otto et al. [2].

Stage	Definition	Anatomy	Haemodynamics
A	At risk of AS	BAV (or other congenital valve anomaly) Aortic valve sclerosis	Aortic V_max_ < 2 m/s with normal leaflet motion
B	Progressive AS	Mild to moderate leaflet calcification/fibrosis of a bicuspid or trileaflet valve with some reduction in systolic motion or Rheumatic valve changes with commissural fusion	Mild AS: aortic V_max_ 2.0–2.9 m/s or mean ∆P < 20 mm Hg Moderate AS: aortic V_max_ 3.0–3.9 m/s or mean ∆P 20–39 mm Hg
C1	Asymptomatic severe AS	Severe leaflet calcification/ fibrosis or congenital stenosis with severely reduced leaflet opening	Aortic V_max_ ≥ 4 m/s or mean ∆P ≥ 40 mm Hg AVA typically is ≤1.0 cm^2^ (or AVA_i_ 0.6 cm^2^/m^2^) but not required to define severe AS Very severe AS is an aortic V_max_ ≥ 5 m/s or mean P ≥ 60 mm Hg
C2	Asymptomatic severe AS with LV systolic dysfunction	Severe leaflet calcification/fibrosis or congenital stenosis with severely reduced leaflet opening	Aortic V_max_ ≥ 4 m/s or mean ∆P ≥ 40 mm Hg AVA typically ≤ 1.0 cm^2^ (or AVA_i_ 0.6 cm^2^/m^2^) but not required to define severe AS
D1	Symptomatic severe high-gradient AS	Severe leaflet calcification/fibrosis or congenital stenosis with severely reduced leaflet opening	Aortic V_max_ ≥ 4 m/s or mean ∆P ≥ 40 mm Hg AVA typically ≤ 1.0 cm^2^ (or AVA_i_ ≤ 0.6 cm^2^/m^2^) but may be larger with mixed AS/AR
D2	Symptomatic severe low-flow, low-gradient AS with reduced LVEF	Severe leaflet calcification/fibrosis with severely reduced leaflet motion	AVA ≤ 1.0 cm^2^ with resting aortic V_max_ < 4 m/s or mean ∆P < 40 mm Hg Dobutamine stress echocardiography shows AVA < 1.0 cm^2^ with V_max_ ≥ 4 m/s at any flow rate
D3	Symptomatic severe low-gradient AS with normal LVEF or paradoxical low-flow severe AS	Severe leaflet calcification/ fibrosis with severely reduced leaflet motion	AVA ≤ 1.0 cm^2^ (indexed AVA ≤ 0.6 cm^2^/m^2^) with an aortic V_max_ < 4 m/s or mean ∆P < 40 mm Hg AND Stroke volume index < 35 mL/m^2^ Measured when patient is normotensive (systolic blood pressure < 140 mm Hg)

**Table 2:** Condition based on the extent of cardiac damage and its impact on all-cause death. AF indicates atrial fibrillation; E/e′ = ratio of early diastolic mitral inflow velocity and mitral annular tissue velocity; LA = left atrial; LAVi: LA indexed volume; LV = left ventricular; LVEF: LV ejection fraction; MR: mitral regurgitation; RV = right ventricular; sPAP: pulmonary artery systolic pressure; TR: tricuspidal regurgitation. Adapted from Genereux et al. [34].

Stage		Echocardiographic Findings	1 Year All-Cause Mortality
0	No Cardiac Damage		4.4%
1	LV Damage	LV mass index > 115 g/m^2^ (M) or >95 m^2^ (F); E/e′ > 14; LVEF < 50%.	9.2%
2	LA or Mitral Damage	LAVi > 34 mL/ m^2^; moderate-severe MR; AF.	14.4%
3	Pulunary Vasculature or Tricuspid Damage	sPAP ≥ 60 mmhg; moderate-severe TR	21.3%
4	RV Damage	Moderate-severe RV dysfunction	24.5%

## Data Availability

No new data were created or analyzed in this study.

## Abbreviations

AS	Aortic Stenosis
SAVR	surgical aortic valve replacement
TAVR	transcatheter aortic valve replacement
BMP-2	bone morphogenetic protein 2
TTE	Transthoracic echocardiography
AVA	aortic valve area
DVI	dimensionless velocity index
SVI	stroke volume index
LVEF	left ventricular ejection fraction
DSE	dobutamine stress echocardiography
TEE	Transoesophageal echocardiography
CPET	Cardiopulmonary exercise testing
CT	computed tomography
CMR	cardiac magnetic resonance
LGE	late gadolinium enhancement
ECV	extracellular volume fraction
ESC	European Society of Cardiology
AVR	Aortic valve replecement
KCCQ	Kansas City Cardiomyopathy Questionnaire
CABG	coronary artery bypass graft
ATTR-CA	transthyretin cardiac amyloidosis
SAPT	single antiplatelet therapy
CS	Cardiogenic shock
LV	Left ventricular
LVEDP	LV end-diastolic pressure
PCWP	post-capillary wedge pressure
CVP	central venous pressure
ADHF	advanced heart failure
BAV	balloon aortic valvuloplasty
AR	aortic regurgitation
VARC	Valve Academic Research Consortium
RCTs	randomized controlled trials
ATTRwt	wild-type ATTR amyloidosis
AVC	aortic valve calcification
